# Hepatic inflammatory pseudotumor associated with primary biliary cholangitis and elevated alpha-fetoprotein lectin 3 fraction mimicking hepatocellular carcinoma

**DOI:** 10.1186/s40792-018-0523-3

**Published:** 2018-09-10

**Authors:** Sho Endo, Yusuke Watanabe, Yuji Abe, Tomohiko Shinkawa, Sadafumi Tamiya, Kazuyoshi Nishihara, Toru Nakano

**Affiliations:** 1 Department of Surgery, Kitakyushu Municipal Medical Center, 2-1-1 Bashaku, Kokurakita-ku, Kitakyushu, 802-0077 Japan; 2 Department of Pathology, Kitakyushu Municipal Medical Center, 2-1-1 Bashaku, Kokurakita-ku, Kitakyushu, 802-0077 Japan

**Keywords:** Hepatic inflammatory pseudotumor, Tumor marker, Alpha-fetoprotein, Lectin 3 fraction

## Abstract

**Background:**

Hepatic inflammatory pseudotumor (IPT) is a rare benign lesion. Because there is no specific laboratory marker or radiographic appearance, the majority of reported cases of hepatic IPT have been diagnosed after surgery or at autopsy. The etiology of hepatic IPT remains unclear but several mechanisms have been postulated such as infection or immune reaction.

**Case presentation:**

A 79-year-old woman had been seeing her family doctor for hypertension, and she had been diagnosed with liver dysfunction for about 10 years. She continued attending follow-ups because of her drinking habit. Two months before her visiting our institution, further elevation of hepatobiliary enzymes was noted, and abdominal ultrasonography showed a hepatic tumor 4 cm in diameter in the lateral segment, so she was referred to our hospital. Hepatocellular carcinoma (HCC) was suspected because alpha-fetoprotein (102 ng/ml) (AFP) and lectin 3 (L3) fraction (85.4%) were elevated and the appearance on enhanced computed tomography was not inconsistent with HCC. Thus, we performed laparoscopic hepatectomy. She recovered uneventfully and was discharged on postoperative day 7. Pathological diagnosis revealed that the tumor was hepatic IPT and that the background liver condition was primary biliary cholangitis (PBC). AFP and L3 fraction decreased to normal ranges after surgery.

**Conclusions:**

In 7 of 29 patients (24.1%) with reported cases of tumor markers in liver IPT, carbohydrate antigen 19-9 was elevated and AFP was elevated in 2 of 58 patients (3.4%). AFP is also frequently elevated in benign liver diseases such as hepatitis and liver cirrhosis, and L3 fraction has been used as a tumor marker for HCC with high specificity. To our knowledge, this is the first report of a case diagnosed with liver IPT in which AFP and L3 fraction increased before surgery and decreased to the normal range after resection. This confirms the rarity of hepatic IPT associated with PBC and elevated AFP and L3 fraction.

## Background

Hepatic inflammatory pseudotumor (IPT) is a rare benign lesion. The etiology of this disease is not well understood, and the course of treatment has not been established yet [1]. Many patients have symptoms associated with inflammation such as fever, abdominal pain, and fatigue, but some patients have no symptoms [1]. It has been reported that leukocytes and C-reactive protein increase in IPT, reflecting an inflammatory response, but there is no specific laboratory marker for this disease [1]. Because there is also no specific radiographic appearance, the majority of reported cases of hepatic IPT have been diagnosed after surgery or at autopsy [2]. Hepatic IPT has been reported to be associated with cholangitis due to, e.g., bacterial and viral infection, immunity and allergic reaction, and primary sclerosing cholangitis, and there are three reports of hepatic IPT cases that were considered to be caused by primary biliary cholangitis (PBC) [3–5]. Surgical resection is carried in over 70% of cases, and relatively good results have been reported [1]. However, some proportion of hepatic IPT become smaller or disappear without treatment, and Sakai et al. suggested that unnecessary surgery should be avoided when diagnosis has been confirmed by needle biopsy [6]. Based on this background, Kaneko et al. reported a case of hepatic IPT resection after observation for a certain period during which the tumor did not shrink [7]. Here, we report the case of a patient who was diagnosed with hepatic IPT associated with PBC and elevated alpha-fetoprotein (AFP) lectin 3 (L3) fraction mimicking hepatocellular carcinoma (HCC).

## Case presentation

A 79-year-old woman had been seeing her family doctor for hypertension and had been diagnosed with liver dysfunction for about 10 years. She continued to attend follow-ups because of her drinking habit. Two months before her visiting our institution, further elevation of hepatobiliary enzymes was noted, and abdominal ultrasonography showed a hepatic tumor of 4 cm in diameter in the lateral segment, so she was referred to our hospital. HCC was suspected because AFP (102 ng/ml) and L3 fraction (85.4%) were elevated, and the appearance on enhanced computed tomography (CT) was not inconsistent with HCC. Thus, she was hospitalized for surgery. The patient was a non-smoker, had a history of habitual alcohol consumption, and reported a medical history of hypertension and hyperlipidemia. Her father and one of her brothers had had esophagus cancers, two of her brothers had liver cirrhosis, and one of her brothers received dialysis.

With regard to complete blood count, platelets decreased to 131,000/μl. Leukocyte elevation and anemia were not observed. Blood biochemistry showed aspartate transaminase, alanine transaminase, alkaline phosphatase, and gamma-glutamyltranspeptidase were elevated to 51 U/l, 42 U/l, 478 U/l, and 136 U/l, respectively. Blood urea nitrogen and creatinine were elevated to 20.2 mg/dl and 1.04 mg/dl, respectively. Total protein, albumin, and bilirubin were within the normal range, and C-reactive protein was not significantly increased. Coagulation was normal, and hepatitis virus tests were negative. The tumor markers AFP and L3 fraction were elevated to 102 ng/ml and 85.4%, respectively. Carcinoembryonic antigen, carbohydrate antigen 19-9 (CA 19-9), and protein induced by vitamin K absence II were within normal limits. Indocyanine green 15 min retention rate was elevated to 16.0%. As for liver fibrosis markers, Mac-2 binding protein glycosylation isomer and type IV collagen 7S were elevated to 2.12 COI and 8.3 ng/ml, respectively. Anti-mitochondrial antibody, immunoglobulin G, and antinuclear antibody were elevated to 1:147, 2093 mg/dl, and 1:320, respectively.

**Fig. 1:**
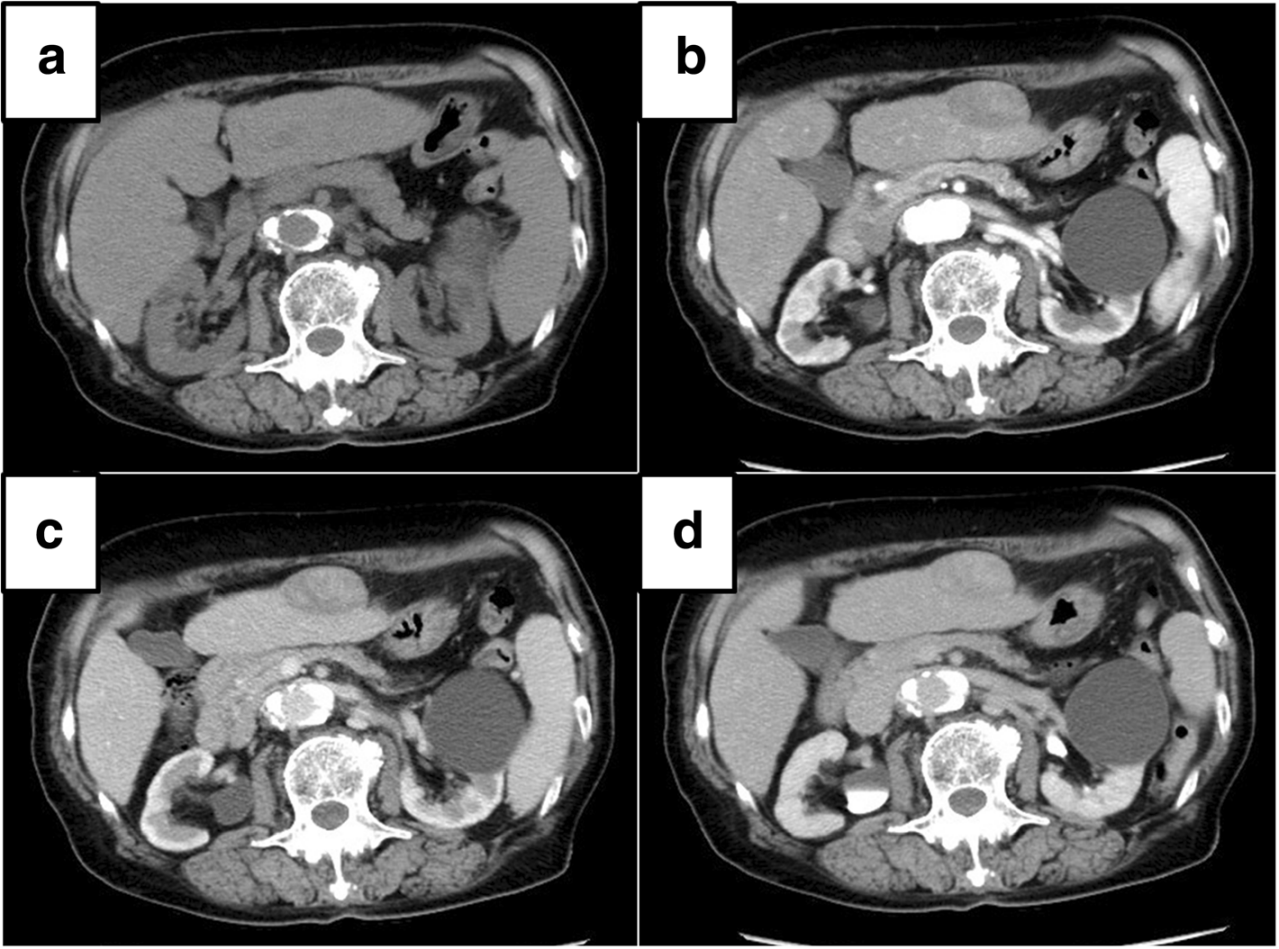
Preoperative contrast computed tomography. Right lobe atrophy, left lobe enlargement, and irregularities on the surface were observed, suggesting liver cirrhosis. A tumor 39 mm in diameter was growing on the outside of the ventral liver segment three. **a** The tumor showed slightly low absorption before contrasting. **b** The tumor showed non-uniform slight contrast in the arterial phase. **c** The tumor showed heterogeneous but mostly lower absorption than the surrounding liver parenchyma in the portal vein phase. **d** The tumor showed equally distributed areas of the same and lower absorption than the surrounding liver parenchyma in the delayed phase

**Fig. 2:**
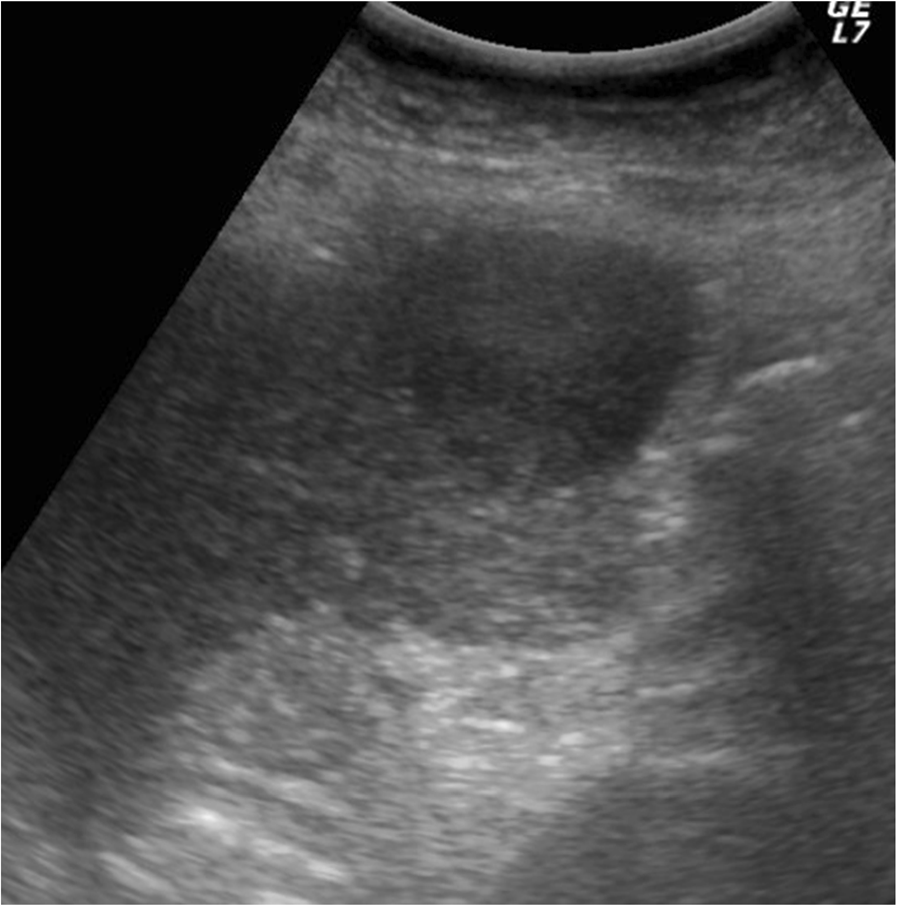
Preoperative ultrasonography. A hypoechoic lesion 34 mm in diameter was observed, and the appearance was not inconsistent with hepatocellular carcinoma

Contrast CT was performed at our institution (Fig. 1). It showed right lobe atrophy, left lobe enlargement, and irregularities on the surface, suggesting liver cirrhosis. A tumor 39 mm in diameter was growing on the outside of the ventral liver segment three. This tumor showed slightly low absorption before contrasting, non-uniform slight contrast in the arterial phase, heterogeneous but mostly lower absorption than the surrounding liver parenchyma in the portal vein phase, and equally distributed areas of the same and lower absorption than the surrounding liver parenchyma in the delayed phase. We considered it likely that this tumor was HCC because of its growth pattern, contrast in the arterial phase, and tumor marker elevation. Non-uniform contrast in the tumor might suggest degeneration or fibrosis. Ultrasonography showed a hypoechoic lesion 34 mm in diameter, the appearance of which was not inconsistent with HCC (Fig. 2). Therefore, laparoscopic hepatectomy was performed under the preoperative diagnosis of HCC without magnetic resonance imaging, positron emission tomography-CT, or reexamination of tumor markers.

When observed with a laparoscope, the yellow tumor was growing on the ventral liver segment three. After the omentum adhering to the tumor was dissected at a sufficient distance from the tumor, we confirmed the tumor by ultrasonography and marked a hepatectomy line 2 cm in diameter around the margin of the tumor edge. The hepatectomy was started from the right caudal side, and the segment three Glisson branch and left hepatic vein were dissected after clipping. After completing the hepatectomy along the marked line, we confirmed that there was no bleeding or bile leak on the hepatectomy surface. A drain was placed in the dorsal side of the hepatectomy surface, and the operation was terminated. The operation time was 2 h and 15 min and the bleeding volume was 1 g.

**Fig. 3:**
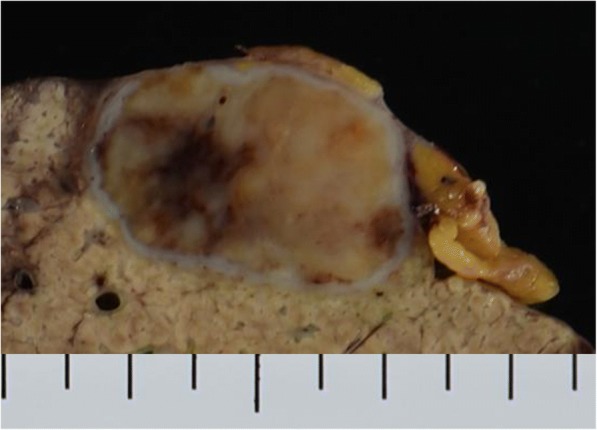
Macroscopic findings of the resected specimen cut surface. The heterogeneous mainly yellow tumor with fibrous white and black parts, accompanied with white capsule around

**Fig. 4:**
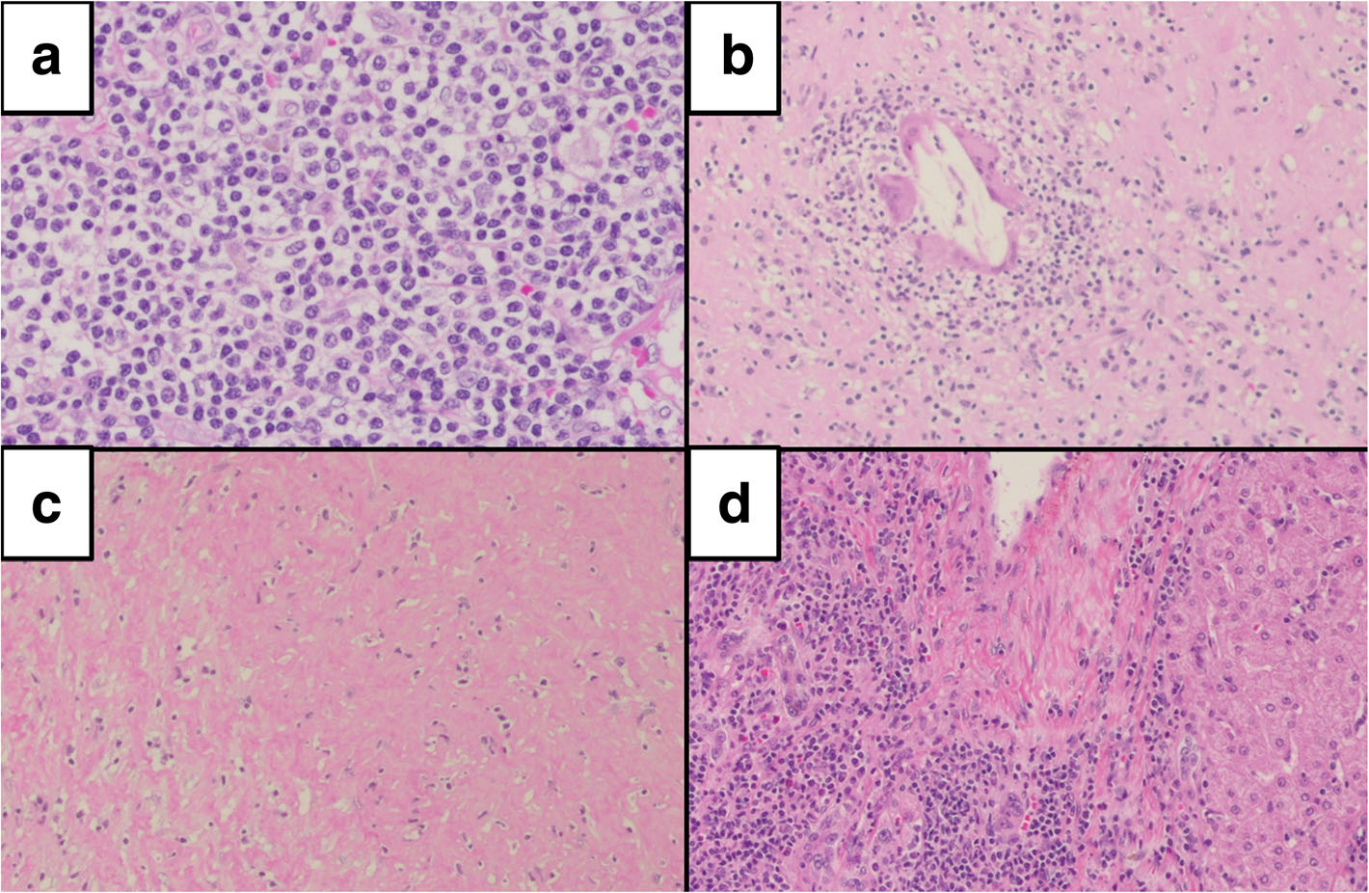
Microscopic findings of the resected specimen. **a** The tumor consisted mainly of collagen fibers, lymphocytes, and plasma cells (× 20). **b** The giant cells contacting cholesterin were observed within the tumor (× 10). **c** The hyalinization was observed within the tumor (× 10). **d** The liver parenchyma outside the tumor area. Expansion of the portal areas was observed as well as crosslinked fibers (× 4)

Macroscopic findings of the resected specimen revealed a heterogeneous and mostly yellow tumor with fibrous white and black parts, accompanied with a white surrounding capsule (Fig. 3). Light microscopy showed that the tumor consisted mainly of collagen fibers, lymphocytes, and plasma cells (Fig. 4a). Cholesterin-containing giant cells (Fig. 4b) and hyalinization (Fig.4c) were also observed. Thus, the tumor was diagnosed as hepatic IPT. In the liver parenchyma outside the tumor area, expansion of the portal area was observed as well as crosslinked fibers (Fig. 4d), chronic non-pyogenic cholangitis, and epithelial cell granulation, suggesting that background liver condition was PBC. All resected specimen tissues were analyzed, but no lesions suggesting tumor existed. Immunochemical staining with anti-AFP antibody was performed, and some hepatocytes around the tumor were stained, but the inside of the tumor did not stain at all.

**Fig. 5:**
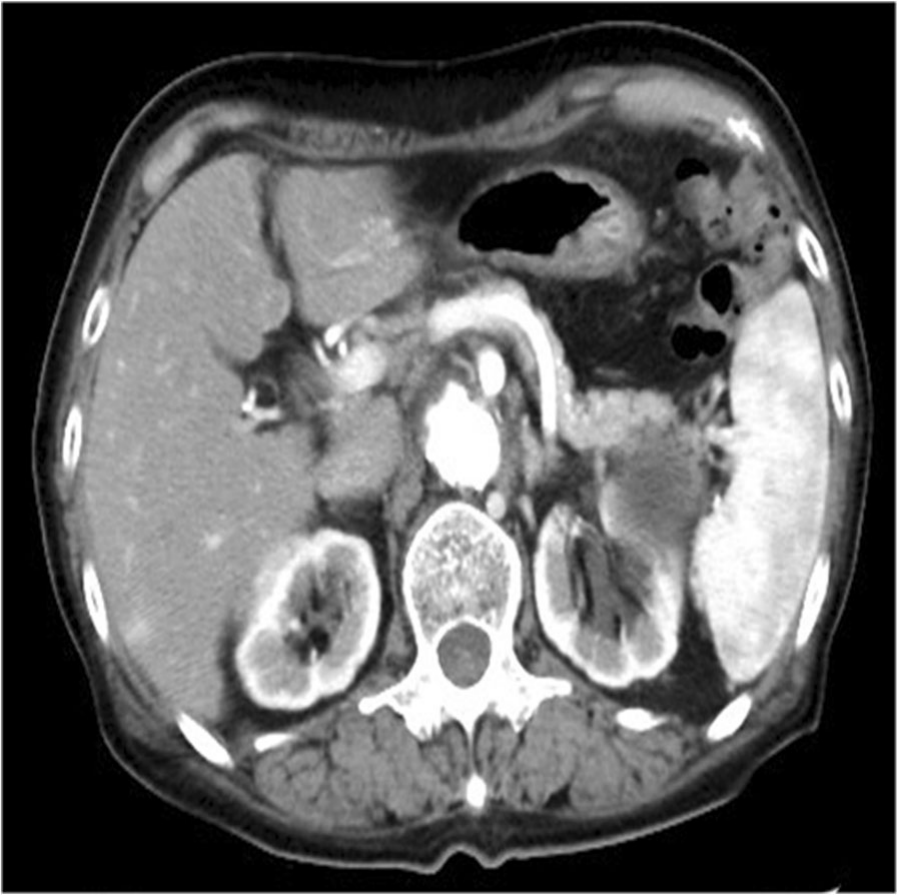
Contrast computed tomography in the arterial phase, performed on postoperative day 36. None of the findings suggested a tumor in the residual liver

Postoperatively, the patient recovered uneventfully and was discharged on postoperative day 7. On postoperative day 36, a blood examination showed that both AFP (4 ng/ml) and L3 fraction (8.4%) had decreased to within normal ranges. None of the findings, including contrast CT performed on the same day, suggested tumor in the residual liver (Fig. 5).

### Discussion

In this case, HCC was strongly suspected because AFP and L3 fraction were elevated. Imaging findings were consistent with HCC, although they were not completely typical. Thus, laparoscopic hepatectomy was performed under the preoperative diagnosis of HCC. However, the liver lesion was ultimately diagnosed as hepatic IPT by histopathology. According to a report on tumor markers in liver IPT, CA 19-9 was elevated in 7 of 29 patients (24.1%) and AFP was elevated in 2 of 58 (3.4%) [8]. AFP is also frequently elevated in benign liver diseases such as hepatitis and liver cirrhosis [9], and L3 fraction has been used as a tumor marker for highly specific HCC [10]. It is also reported that a high percentage of L3 fraction significantly correlates with low survival rate after HCC treatment [10]. Thus, the significance of L3 fraction as a marker for biological malignancy has been drawing attention.

In this case, although the pathological diagnosis was liver IPT, the AFP and L3 fractions were elevated before resection, and both markers postoperatively decreased to the normal ranges. This prompted us to consider the possibility of spontaneous regression of HCC, so we investigated all resected specimen tissues, but no other tumor components were found. AFP L3 fraction is also elevated in some cases of acute hepatitis or chronic active hepatitis [11, 12], and it is further elevated in fulminant hepatic failure [13, 14]. Additionally, it is elevated in acute liver injury, including acute-onset autoimmune hepatitis and acute liver failure [15]. These data suggested AFP L3 fraction may reflect liver regeneration. With regard to tumors, it is reported that elevated AFP L3 fraction has been observed in multiple pancreatic acinar cell carcinoma patients [16], but to our knowledge, there have been no reports of elevated L3 fraction being decreased by resection of liver tumors other than HCC, including cholangiocarcinoma and IPT. Considering that L3 fraction can be elevated by severe acute hepatitis, elevation of the L3 fraction in this case could be caused by local intense inflammation within the tumor. However, we cannot explain the detailed mechanism of this, and future research to improve the understanding of the pathology and other aspects of liver IPT is expected.

## Conclusions

We reported a rare hepatic IPT associated with PBC and elevated AFP and L3 fraction.

## Abbreviations

AFP: Alpha-fetoprotein
CA 19-9: Carbohydrate antigen 19-9
CT: Computed tomography
HCC: Hepatocellular carcinoma
IPT: Inflammatory pseudotumor
L3 fraction: Lectin 3 fraction
PBC: Primary biliary cholangitis
